# Supplementary material for: Bioengineered amyloid peptide for rapid screening of inhibitors against main protease of SARS-CoV-2
Source: Nat Commun. 2024 Mar 7;15:2108. doi: 10.1038/s41467-024-46296-7 (PMC10920794; doi:10.1038/s41467-024-46296-7)
Supplement: Supplementary file 1 — Supplementary Information [file 41467_2024_46296_MOESM1_ESM.pdf]

Supplementary Information

Bioengineered amyloid peptide for rapid screening of inhibitors against main protease of SARS-CoV-2

Dongtak Lee[1,2,3#], Hyo Gi Jung[1,4,#], Dongsung Park[1,5,#], Junho Bang[1,4], Da Yeon Cheong[6,7], Jae Won Jang[1,4], Yonghwan Kim[1,4], Seungmin Lee[1,8], Sang Won Lee[1,9], Gyudo Lee[6,7], Yeon Ho Kim[1,4], Ji Hye Hong[1, 8], Kyo Seon Hwang[5,*], Jeong Hoon Lee[8,*], Dae Sung Yoon[1,4,10,*]

[1] School of Biomedical Engineering, Korea University, Seoul 02841, South Korea

[2] Center for Nanomedicine, Department of Anesthesiology, Perioperative and Pain Medicine, Brigham and Women's hospital, Boston, MA 02115, USA

[3] Harvard Medical School, Boston, MA, 02115, USA

[4] Interdisciplinary Program in Precision Public Health, Korea University, Seoul 02841, South Korea

[5] Department of Clinical Pharmacology and Therapeutics, College of Medicine, Kyung Hee University, Seoul 02447, South Korea

[6] Department of Biotechnology and Bioinformatics, Korea University, Sejong 30019, South Korea

[7] Interdisciplinary Graduate Program for Artificial Intelligence Smart Convergence Technology, Korea University, Sejong 30019, South Korea

[8] Department of Electrical Engineering, Kwangwoon University, Seoul 01897, South Korea

[9] Terasaki Institute for Biomedical Innovation, Los Angeles, CA, 91367, USA

[10] Astrion Inc, Seoul 02841, South Korea

* Corresponding authors: D.S.Y. (dsyoon@korea.ac.kr); J.H.L. (jhlee@kw.ac.kr); K.S.H. (k.hwang@khu.ac.kr)

[#] These authors contributed equally to this work

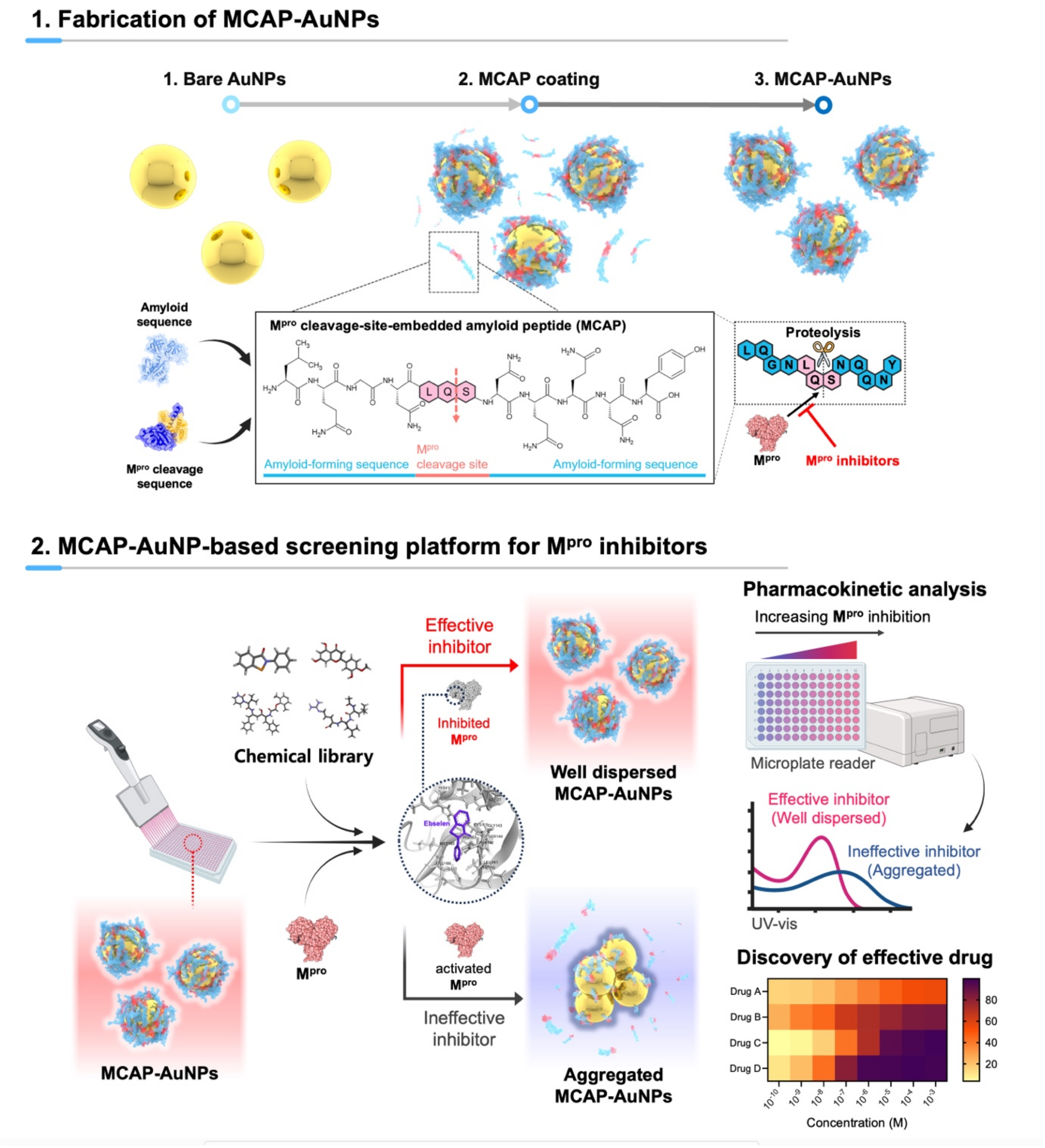

Supplementary Fig 1. Schematic illustration of the MCAP-AuNPs-based drug screening platform.

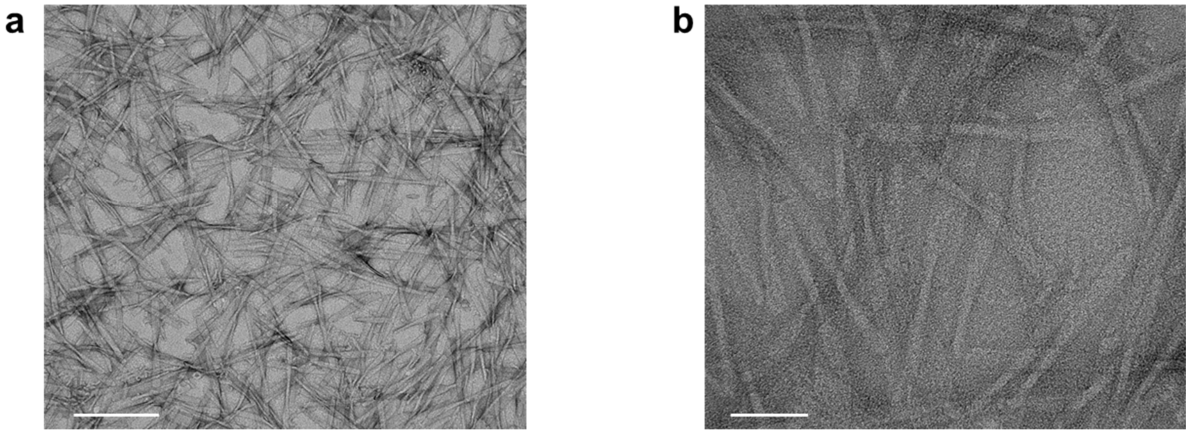

Supplementary Fig. 2. TEM images of the MCAP fibril. The size of the white scale bar is (a) 200 nm, and (b) 50 nm, respectively. The persistence length of MCAP fibrils was 229.95 ± 94.22 nm which was analyzed by Easy Warm software. The TEM images were measured at least three times.

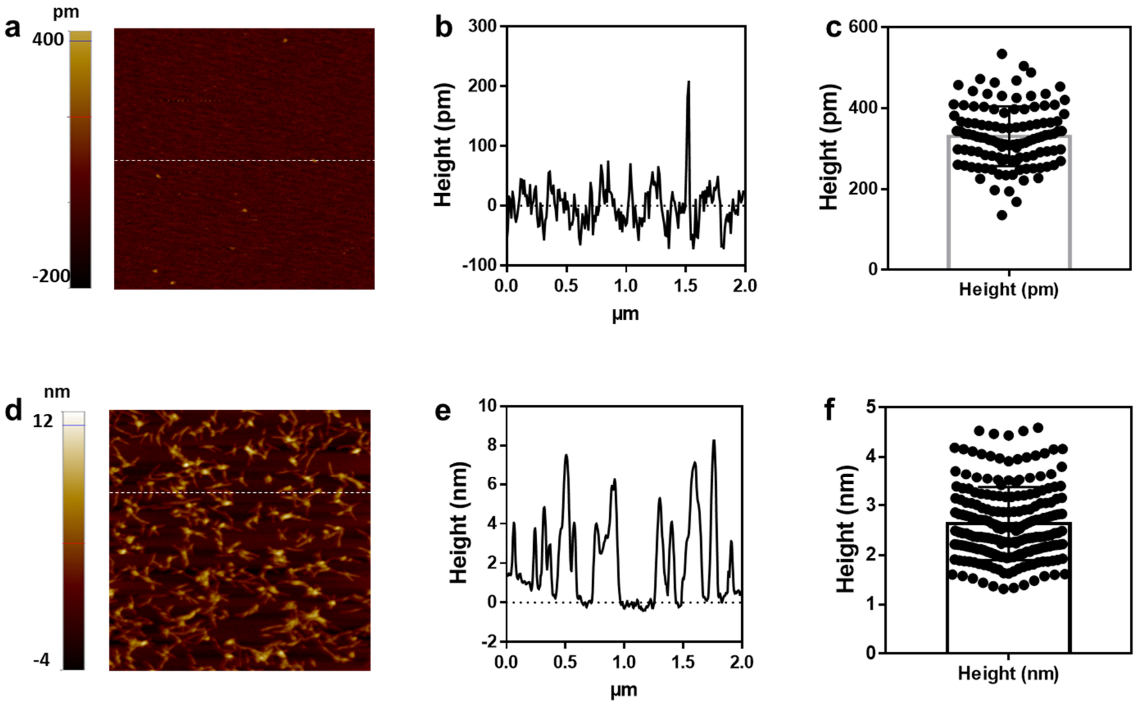

Supplementary Fig. 3. AFM analysis of the MCAP monomers and fibrils. (a) AFM image (4 × 4 μm^2) of the MCAP monomers. (b) The cross-sectional profile of the MCAP monomers along the white dash line in (a). (c) The height of the MCAP monomers (0.33 ± 0.07 nm) was measured by AFM. Data are presented as mean values ± standard deviation (SD) from n=113 independent samples. (d) AFM image (4 × 4 μm^2) of the MCAP fibrils. (e) The cross-sectional profile of the MCAP monomers along the white dash line in (d). (f) The height of the MCAP fibrils (2.64 ± 0.74 nm) was measured by AFM. Data are presented as mean values ± standard deviation (SD) from n=204 independent samples. The AFM images were measured at least three times. The average height of the MCAP monomer and fibril was analyzed by NX10 software (Park Systems, South Korea).

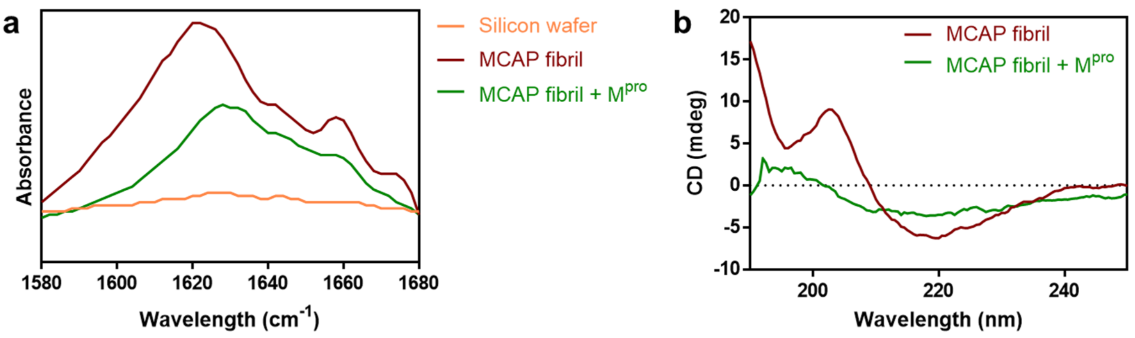

Supplementary Fig. 4. FT-IR and CD analysis. (a) FT-IR analyses of the MCAP fibrils and the M^{pro}-treated MCAP fibrils. The absorbance peak of MCAP fibrils near 1620 cm^{-1} wavelength and 1660 cm^{-1} wavelength represent the β-sheet structure of MCAP fibrils. Note that these peak of MCAP fibrils was notably reduced in the presence of M^{pro}, indicating the proteolytic activity of M^{pro} toward MCAP fibrils. (b) CD spectra of the MCAP fibrils and the M^{pro}-treated MCAP fibrils. Note that the M^{pro} decreased the peak of the β-sheet structure (190 to 220 nm) of CD spectra.

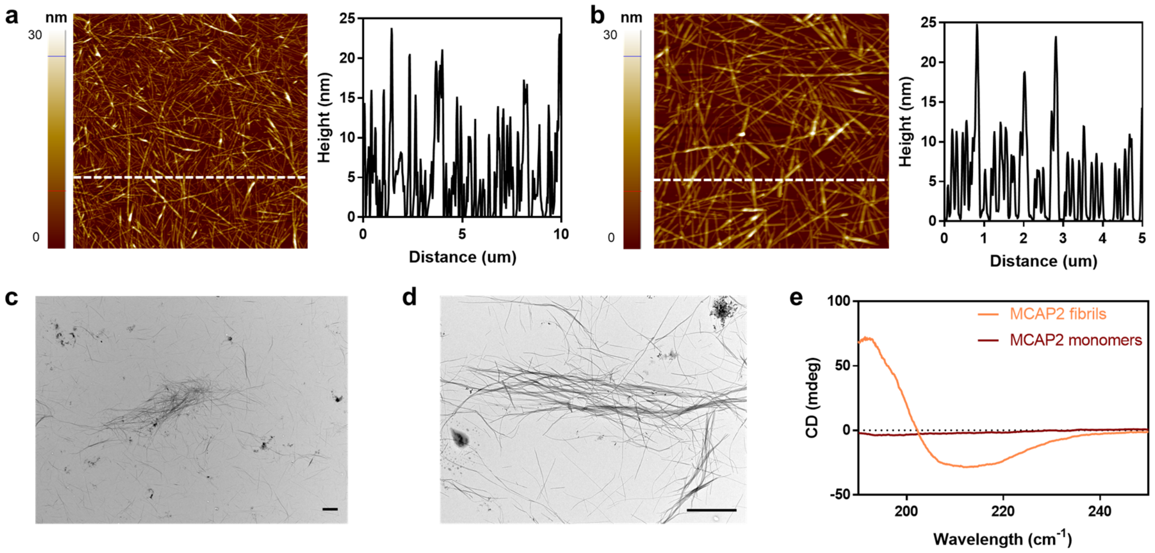

Supplementary Fig. 5. Amyloidogenicity assay of the MCAP2 fibrils. (a) and (b) Topological analysis of the MCAP2 fibrils by AFM. The height map image (left) of MCAP2 fibrils, and the topographic cross-sectional profile (right) are taken through the white dashed line of the height map image. The image sizes are (a) 10 x 10 µm and (b) 5 x 5 µm, respectively. (c) and (d) TEM images of the MCAP2 fibrils. The black scale bar in TEM images is 2 µm in width. Note that both AFM analysis and TEM images demonstrated the amyloid-forming properties of MCAP2. (e) CD spectra of the MCAP2 fibrils and monomers. Note that the β-sheet structure of MCAP2 fibrils was observed by CD analysis with peaks near 210 cm^{-1} and 190 cm^{-1}. The AFM and TEM images were measured at least three times.

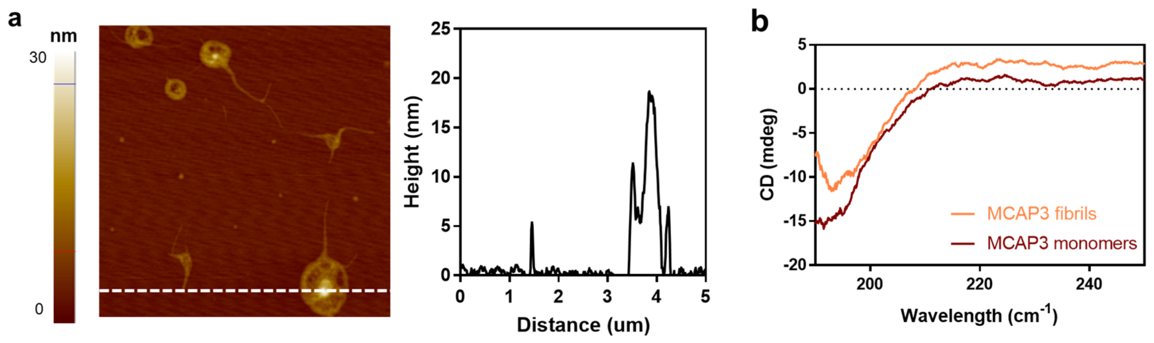

Supplementary Fig. 6. Amyloidogenicity assay of MCAP3 fibrils. (a) Topological analysis of the MCAP3 fibrils by AFM. The height map image (left) of MCAP3 fibrils, and the topographic cross-sectional profile (right) are taken through the white dashed line of the height map image. Note that the AFM analysis of MCAP3 aggregates showed an absence of a fibril-like structure. The AFM images were measured at least three times. (b) CD spectra of the MCAP3 fibrils and monomers. The CD spectra of MCAP3 aggregates exhibited a peak near 190 cm^{-1}, representing the random coil structure. Note that these observations indicate that MCAP3 aggregates are not in the structural form of fibril fibers and have no amyloidogenicity compared to MCAP and MCAP2.

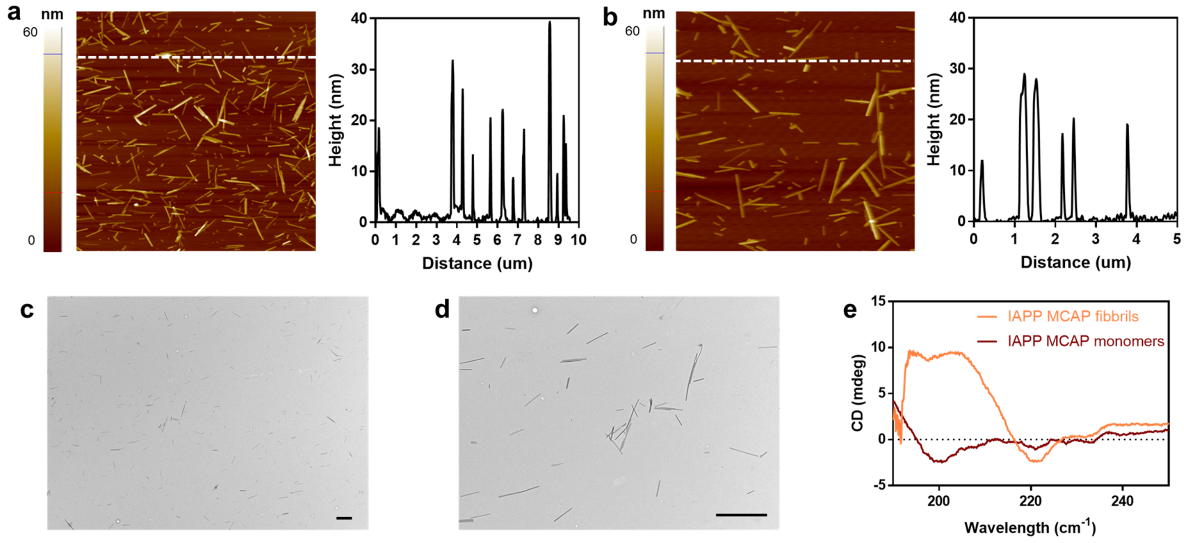

Supplementary Fig. 7. Amyloidogenicity assay of the IAPP MCAP fibrils. (a) and (b) Topological analysis of IAPP fibrils by AFM. The height map image (left) of IAPP fibrils, and the topographic cross-sectional profile (right) taken through the white dashed line of the height map image. The images are (a) 10 x 10 µm and (b) 5 x 5 µm in size, respectively. (c) and (d) TEM images of the IAPP fibrils. The black scale bar in each TEM image is 2 µm in width. The AFM and TEM images were measured at least three times. (e) CD spectra of the IAPP MCAP fibrils and monomers. The CD spectra for IAPP MCAP fibrils revealed distinctive peaks near 220 cm^{-1} and 190 cm^{-1}, representing Aβ-sheet structure. Note that even after inserting the M^{pro} cleavage sequence (LQS) into the amyloid-forming sequence (SNNFGAIL), it retains amyloidogenicity.

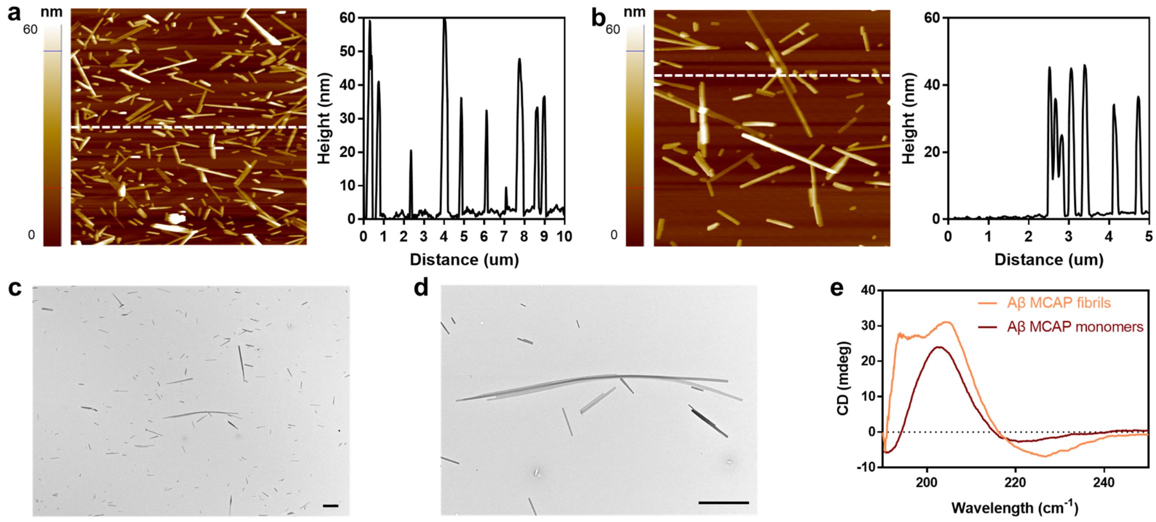

Supplementary Fig. 8. Amyloidogenicity assay of the Aβ MCAP1fibrils. (a) and (b) Topological analysis of the Aβ MCAP1 fibrils by AFM. The height map image (left) of Aβ MCAP1 fibrils, and the topographic cross-sectional profile (right) taken through the white dashed line of the height map image. The images are (a) 10 x 10 μm and (b) 5 x 5 μm in size, respectively. (c) and (d) TEM images of the Aβ MCAP1 fibrils. The black scale bar in each TEM image is 2 μm in width. The AFM and TEM images were measured at least three times. (e) CD spectra of the Aβ MCAP1 fibrils and monomers. The CD spectra of Aβ MCAP1 fibrils have peaks near 220 cm^{-1} and 190 cm^{-1}, representing that they have a β-sheet structure. Note that even after inserting the M^{pro} cleavage sequence (LQS) into the amyloid-forming sequence (KLVFFAE), it retains amyloidogenicity.

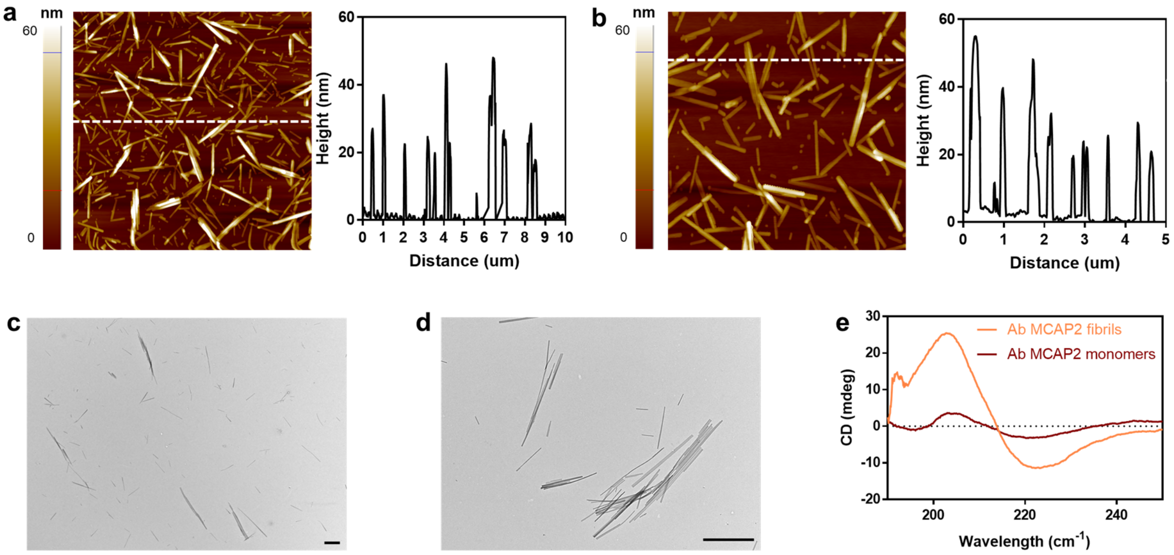

Supplementary Fig. 9. Amyloidogenicity assay of the Aβ MCAP2 fibrils. (a) and (b) Topological analysis of the Aβ MCAP2 fibrils by AFM. The height map image (left) of Aβ MCAP2 fibrils, and the topographic cross-sectional profile (right) are taken through the white dashed line of the height map image. The images are (a) 10 x 10 μm and (b) 5 x 5 μm in size, respectively. (c) and (d) TEM images of the Aβ MCAP2 fibrils. The black scale bar in each TEM image is 2 μm in width. The AFM and TEM images were measured at least three times. (e) CD spectra of the Aβ MCAP2 fibrils and monomers. The CD spectra of Aβ MCAP2 fibrils have peaks near 220 cm^{-1} and 190 cm^{-1}, representing that they have a β-sheet structure. Note that even after inserting the M^{pro} cleavage sequence (LQS) into the amyloid-forming sequence (GGVVIA), it retains amyloidogenicity.

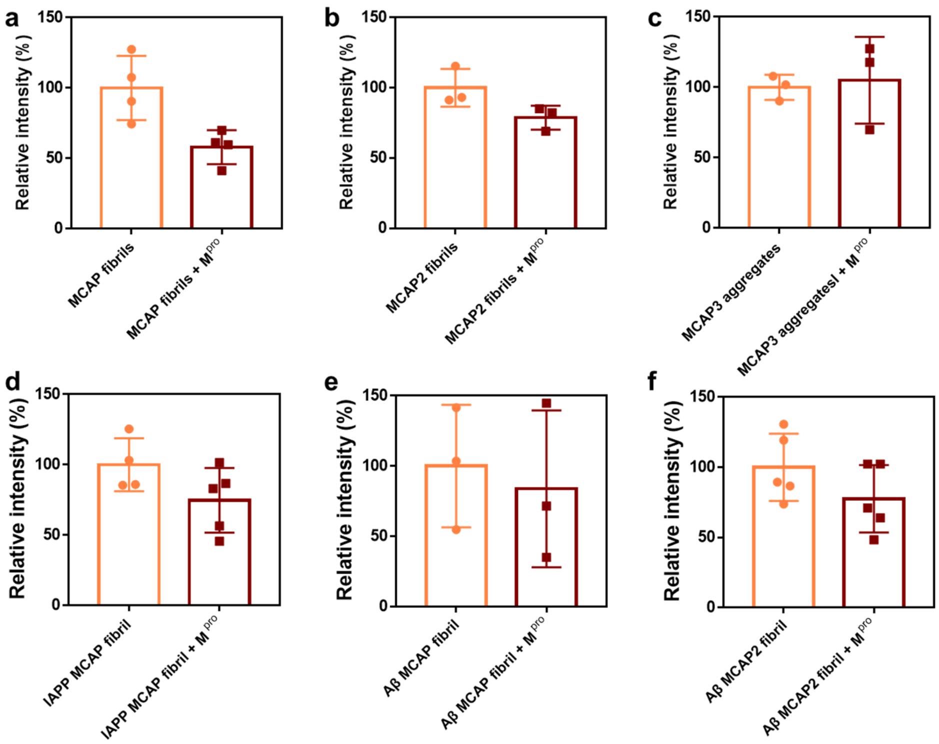

Supplementary Fig. 10. ThT analysis of engineered amyloid fibrils. The ThT intensity changes of (a) the MCAP fibrils, (b) the MCAP2 fibrils, (c) the MCAP3 fibrils, (d) the IAPP MCAP fibrils (e) the Aβ MCAP1 fibrils, and (f) the Aβ MCAP2 fibrils when reacting with M^{pro}. Each sample (100 µM, 80 µL) was pretreated with 20 µl of M^{pro} solution (1.48 µM) for 4h. After that, each fibril was reacted with 20 µM of ThT solution for 1 hour. The ThT fluorescence intensities were measured by excitation at 444 nm and emission at 510 nm. Note that the MCAP, the MCAP2, the IAPP MCAP, the Aβ MCAP1, and the Aβ MCAP2 fibrils reduced 42%, 22%, 26%, 17%, and 23% in ThT fluorescence intensity by M^{pro}, however, the MCAP3 aggregates exhibited no significant signal change upon reaction with M^{pro}. All data are presented as mean values ± SD from independent experiments (MCAP fibril: n=4, MCAP2 fibril: n=3, MCAP3 aggregates: n=3, IAPP MCAP fibril: n=5, Aβ MCAP fibril: n=3, Aβ MCAP2 fibril: n=5).

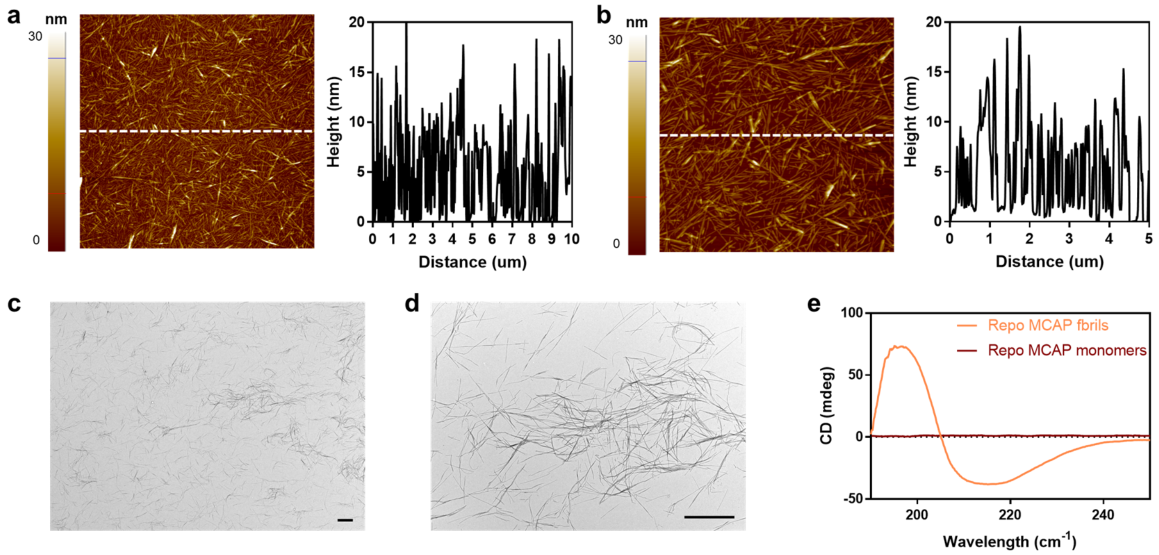

Supplementary Fig. 11. Amyloidogenicity assay of the Repositioned MCAP fibrils. (a) and (b) Topological analysis of the Repositioned MCAP fibrils by AFM. The height map image (left) of Repositioned MCAP fibrils, and the topographic cross-sectional profile (right) taken through the white dashed line of the height map image. The image sizes are (a) 10 x 10 µm and (b) 5 x 5 µm, respectively. (c) and (d) TEM images of the Repositioned MCAP fibrils. The AFM and TEM images were measured at least three times. The black scale bar in TEM images is 2 µm in width. (e) CD spectra of the Repositioned MCAP fibrils and monomers. The CD spectra of the Repositioned MCAP fibrils showed peaks near 220 cm^{-1} and 190 cm^{-1}, indicative of a β-sheet structure. Note that engineered peptides wherein the M^{pro} cleavage sequence (LQS) is attached to the end of amyloid-forming sequences have amyloidogenicity.

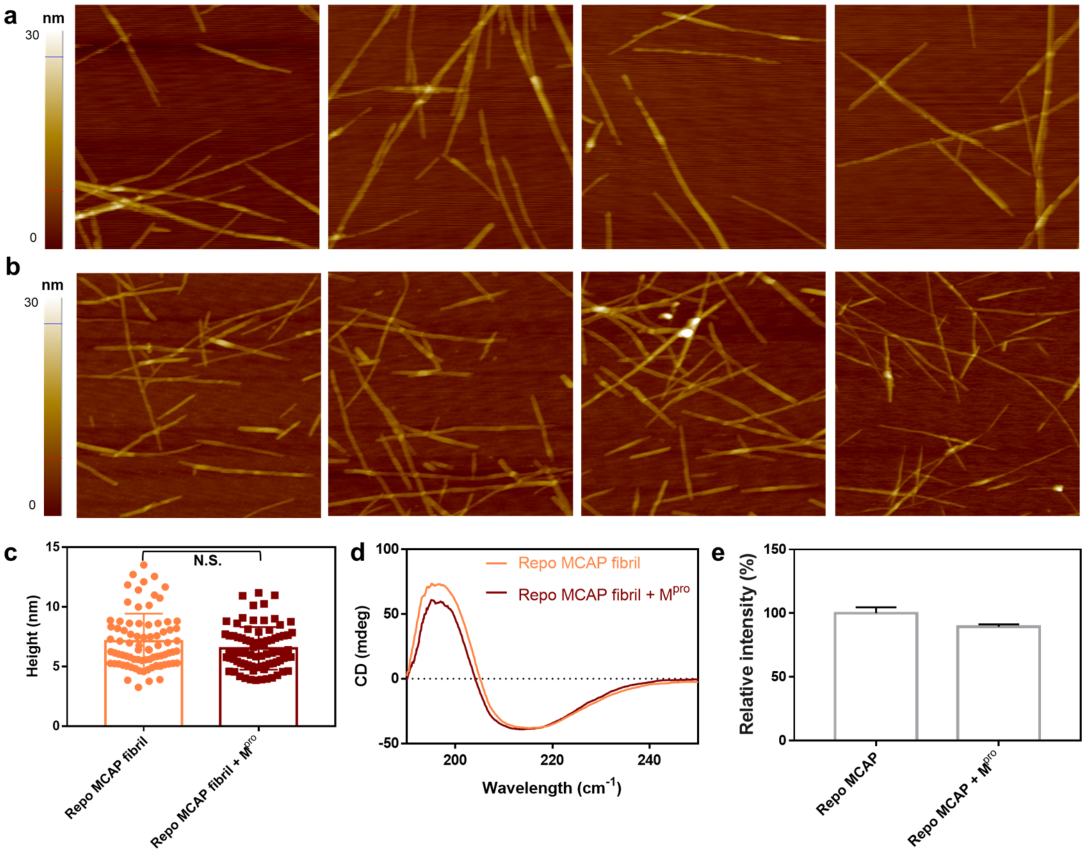

Supplementary Fig. 12. M^{pro} reactivity test of the Reposition MCAP fibrils. (a) Topological analysis of the Repositioned MCAP fibrils by AFM. (b) Topological analysis of the M^{pro}-treated Repositioned MCAP fibrils by AFM. Note that fibrillar structure remained regardless of M^{pro} treatment, indicating that the Repositioned MCAP fibrils did not react with M^{pro}. The AFM images were measured at least three times. (c) Height analysis of the Repositioned MCAP fibrils with/without M^{pro} treatment. Note that the height of the Repositioned MCAP fibrils treated with M^{pro} revealed no significant changes compared to non-treated. Data are presented as mean values ± SD from n=80 independent samples. The height of each fibril was measured by NX10 software (Park Systems, South Korea). (d) CD spectra of the Repositioned MCAP fibrils with/without M^{pro} treatment. Note that the CD spectra of the Repositioned MCAP fibrils reacted with M^{pro} exhibited no peak change compared with the Repositioned MCAP fibrils, indicating the β-sheet structures remained in the presence of M^{pro}. (e) The ThT intensities of the Repositioned MCAP fibrils and the M^{pro}-treated Repositioned MCAP fibrils. The ThT assay of the Repositioned MCAP fibrils treated with M^{pro} was reduced by 10%. Note that the Repositioned MCAP fibrils have no M^{pro} reactivity because even though the M^{pro} retains the cleavage activity at the end of Repositioned MCAP, the full sequence of GNNQQNY is still preserved after the M^{pro} reaction, resulting in no degradation of the fibrillar structure. Data are presented as mean values ± SD from n=3 independent experiments.

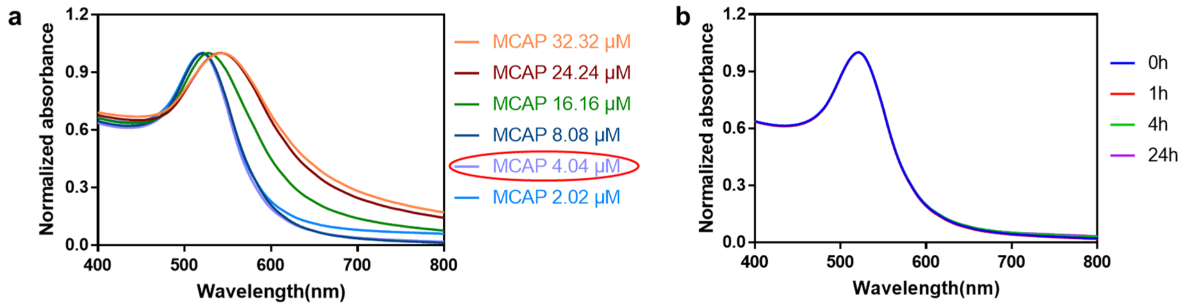

Supplementary Fig. 13. Optimization of the MCAP monomer concentration for fabricating the MCAP-AuNP. (a) UV-vis spectra of MCAP-AuNP solution in PBS after 24h depending on the MCAP monomer concentration. Note that at a low concentration of the MCAP monomer under 4.04 μM, the UV-spectra of MCAP-AuNP were shifted because the MCAP monomers did not fully cover the surface of AuNP. And at a high concentration of the MCAP monomer over 4.04 μM, the red shift of UV-spectra was caused by highly fibrillar MCAP aggregates which induce the aggregation of particles. (b) Time-dependent UV-vis spectra of MCAP-AuNP solution in PBS, which was fabricated by 4.04 μM of MCAP monomer solution. Note that this monomer concentration is appropriate for making the MCAP-AuNP and the MCAP-AuNP is highly stable under physiological salt conditions.

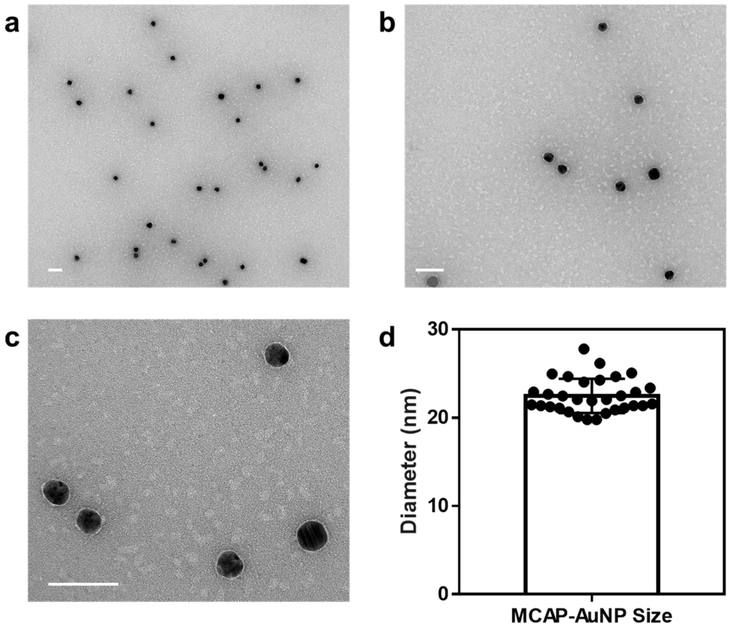

Supplementary Fig. 14. TEM images of MCAP-AuNPs. (a) to (c) TEM images of the MCAP-AuNPs. Note that we analyzed fifty MCAP-AuNPs and observed that 98% of MCAP-AuNPs had a protein corona on the surface of AuNP. The size of white scale bar is 50 nm. The TEM images were measured at least three times. (d) Size distribution of the MCAP-AuNPs. The average diameter of MCAP-AuNPs is measured as 22.47 ± 1.90 nm. The diameter of each MCAP-AuNP was measured by Image J software in TEM images. Data are presented as mean values ± SD from n=31 independent samples.

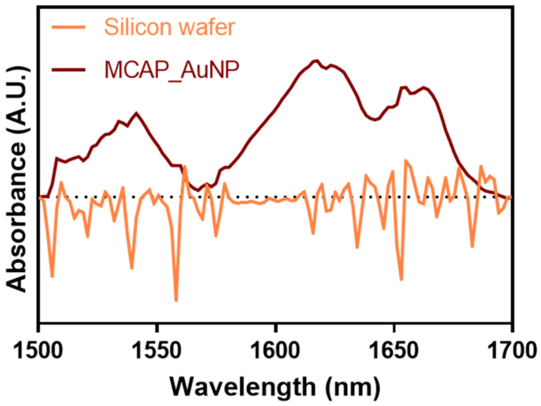

Supplementary Fig. 15. FT-IR spectra of the MCAP-AuNPs on silicon and the silicon wafer only. Prior to the FT-IR analysis, we removed the MCAP aggregates in the supernatant of the MCAP-AuNP solution by centrifugation and deposited MCAP-AuNPs onto a silicon wafer. The MCAP-AuNPs was deposited on the surface of the silicon wafer, and then measured by FT-IR spectrometer. The scanning range spans 1500-1700 cm^{-1} in wavelength and the spectra was 8 nm in resolution. Note that the FT-IR spectra of MCAP-AuNP have β-sheet structure peaks near 1620 nm^{-1} and 1660 nm^{-1}, indicating that MCAP aggregates attached to the MCAP-AuNP have the β-sheet conformation.

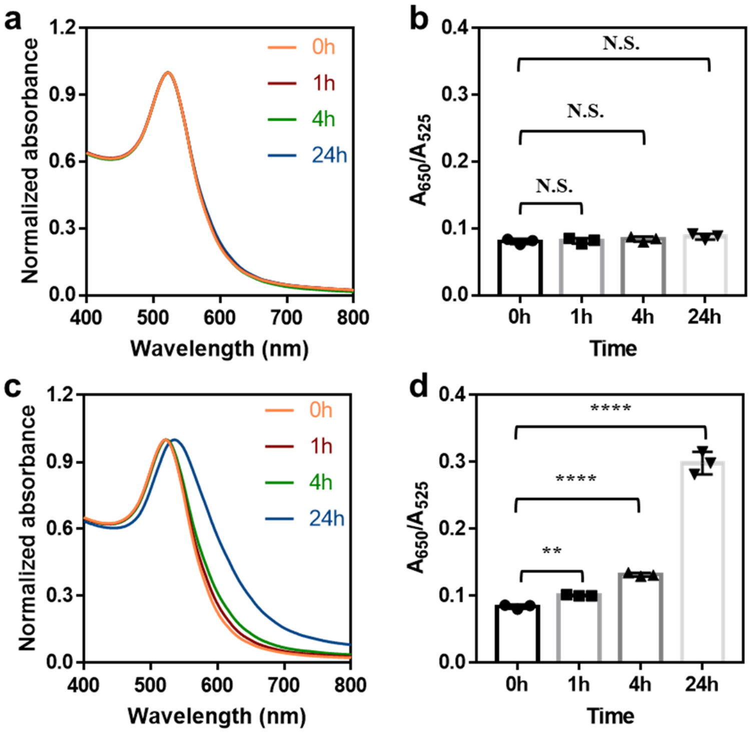

Supplementary Fig. 16. Temperature-dependent stability test of the MCAP-AuNPs. (a) UV-vis spectra of the MCAP-AuNPs at 40 °C depending on time. (b) Relative absorbances (A_{650}/A_{525}) of the MCAP-AuNPs at 40 °C with time. Data are presented as mean values ± SD from n=3 independent experiments. (c) UV-vis spectra of the MCAP-AuNPs at 60 °C depending on time. (d) Relative absorbances (A_{650}/A_{525}) of the MCAP-AuNPs at 60 °C with time. The relative absorbance (A_{650}/A_{525}) of MCAP-AuNP at 60 °C for 24h changed by 71.83% compared to the initial condition, in contrast, MCAP-AuNP at 40 °C showed a negligible change. Note that the MCAP-AuNPs were stable at 40 °C but unstable at the 60 °C conditions due to thermal denaturation. Accordingly, there is no problem in the use of MCAP-AuNPs at the temperature range of 30~40 °C that corresponds to the physiological temperature range of M^{pro}. Data are presented as mean values ± SD from n=3 independent experiments. (N.S: $p \geq 0.05$, *: $p<0.05$, **: $p<0.01$, ***: $p<0.001$, ****: $p<0.0001$).

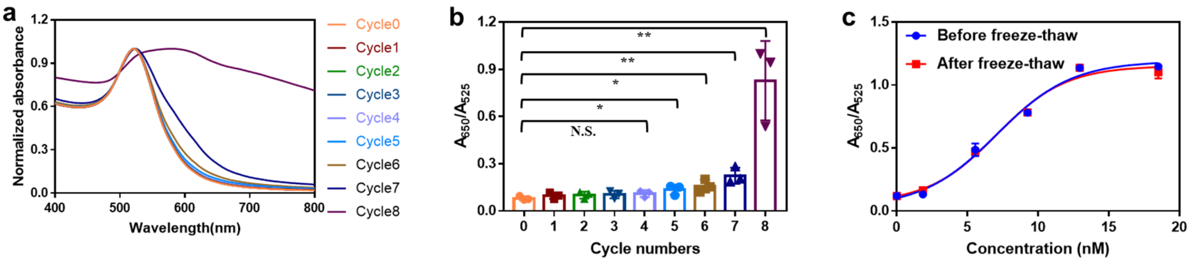

Supplementary Fig. 17. Repeated freeze-thaw test of the MCAP-AuNPs. (a) UV-vis spectra of the MCAP-AuNPs depending on freeze-thaw cycle. The MCAP-AuNP solutions were frozen in the deep freezer at – 80 °C and thawed at room temperature. (b) Relative absorbances (A_{650}/A_{525}) of the MCAP-AuNPs depending on freeze-thaw cycle. Note that UV-vis spectra and relative absorbance (A_{650}/A_{525}) remained unchanged until the fourth freeze-thaw cycle. Starting from the fifth cycle, UV-vis spectra gradually exhibited a red shift, with a dramatic change observed in the eighth cycle. Data are presented as mean values ± SD from n=3 independent experiments. (c) The values of A_{650}/A_{525} of the MCAP-AuNPs were measured before and after M^{pro} reaction depending on the concentration of M^{pro}. Note that the MCAP-AuNPs are suitable for cryopreservation maintaining a constant reactivity by M^{pro} after thawing for use. Data are presented as mean values ± SD from n=3 independent experiments. The freeze-thawed MCAP-AuNPs are marked in red and the normal MCAP-AuNPs are marked in blue. Error bars are not shown where they are smaller than the circle symbols. (N.S: $p \geq 0.05$, *: $p<0.05$, **: $p<0.01$).

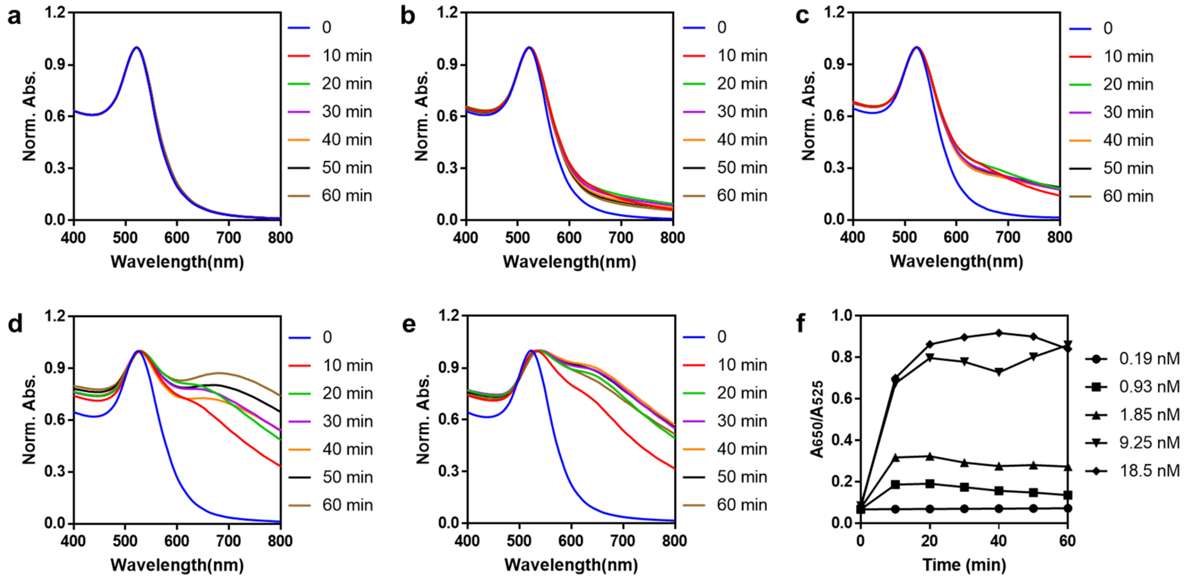

Supplementary Fig. 18. Time-dependent proteolytic analysis of the M^{pro} using the MCAP-AuNP. The time-dependent UV-vis spectra of MCAP-AuNP solution for the various concentrations of the M^{pro}; (a) 0.19 nM, (b) 0.93 nM, (c) 1.85 nM, (d) 9.25 nM, and (e) 18.5 nM. The red shift of UV-spectra was increased over time at each concentration of the M^{pro} (> 0.19 nM) due to the proteolytic activity of the M^{pro}. The degree of the red shift of UV-vis spectra was increased as the M^{pro} concentration increased. (f) The time-dependent relative absorbance (A_{650}/A_{525}) of MCAP-AuNP solution was measured depending on the M^{pro} concentration. Note that the MCAP-AuNP-based platform is suitable for measuring the proteolytic activity of M^{pro}. Data are presented as point values from n=1.

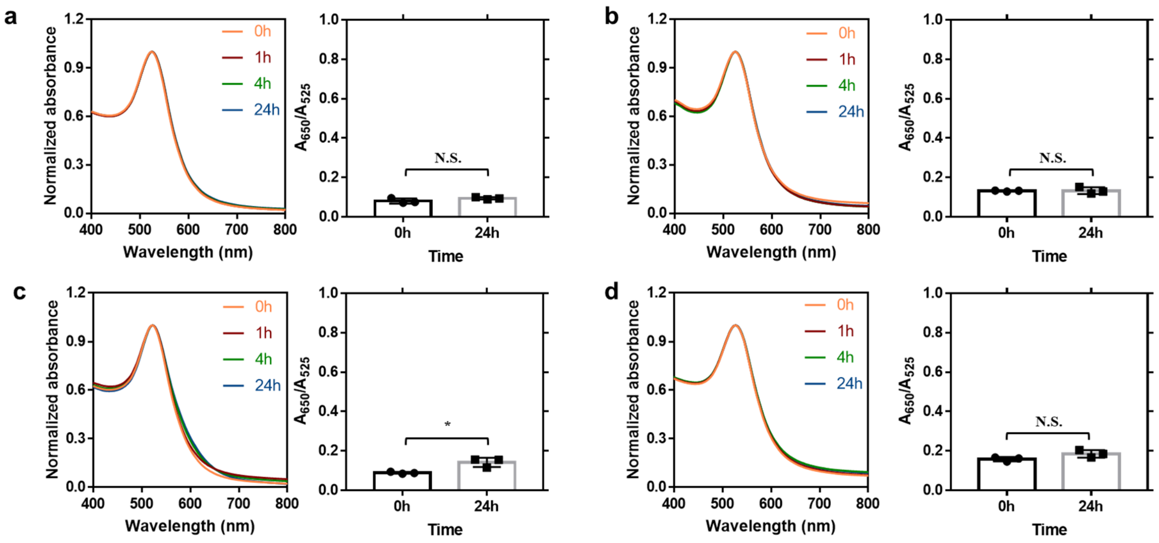

Supplementary Fig. 19. Stability test of the MCAP-AuNPs with biomolecules. The reaction concentrations of each molecule were chosen by physiological range. The left graphs showed the UV-vis spectra of MCAP-AuNPs reacted with (a) 1 $mg \cdot mL^{-1}$ of BSA, (b) 30 $mg \cdot mL^{-1}$ of HSA, (c) 1 $mg \cdot mL^{-1}$ of glucose, and (d) 10 $mg \cdot mL^{-1}$ of IgG depending on time. The right graphs showed the values of A_{650}/A_{525} of the MCAP-AuNPs reaction with biomolecules after 24h. The MCAP-AuNP solution was reacted with biomolecules (BSA, HSA, glucose, and IgG) at room temperature for 24h. Note that the MCAP-AuNPs are not affected by other interfering molecules abundant in physiological conditions and selectively react with M^{pro}. All data in bar graphs are presented as mean values ± SD from n = 3 independent experiments. (N.S: $p \geq 0.05$, *: $p<0.05$, **: $p<0.01$, ***: $p<0.001$).

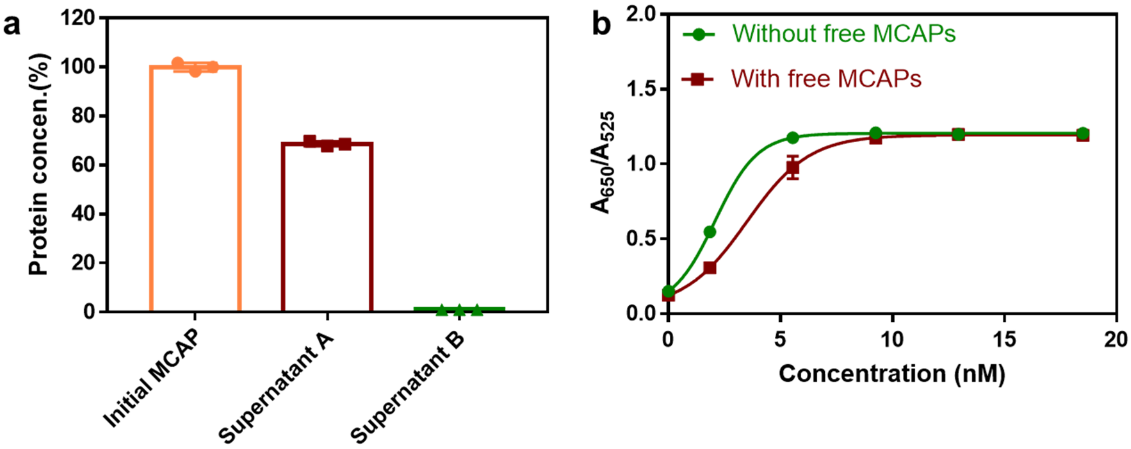

Supplementary Fig. 20. Free MCAP aggregates test of MCAP-AuNP. (a) Relative MCAP concentrations of the MCAP-AuNP solutions before (Supernatant A) and after purification (Supernatant B) compared to the initial MCAP concentration (Initial MCAP). MCAP monomers were used to make a protein standard curve by BCA protein assay (Thermo Fisher Scientific, USA) to measure MCAP concentration. The MCAP concentration in Supernatant A was found to be 68.7% compared to the initial MCAP concentration. In contrast, the MCAP concentration of the supernatant B (after centrifugation) was negligible. (b) Sigmoidal dose-response curves as a function of M^{pro} concentration for the MCAP-AuNP solution in the presence and absence of free MCAPs. Note that the half-maximal effective concentration (EC_{50}) was slightly shifted from 3.5 nM to 2.1 nM in the presence of free MCAPs. All data are presented as mean values ± SD from n = 3 independent experiments. Error bars are not shown where they are smaller than the circle symbols.

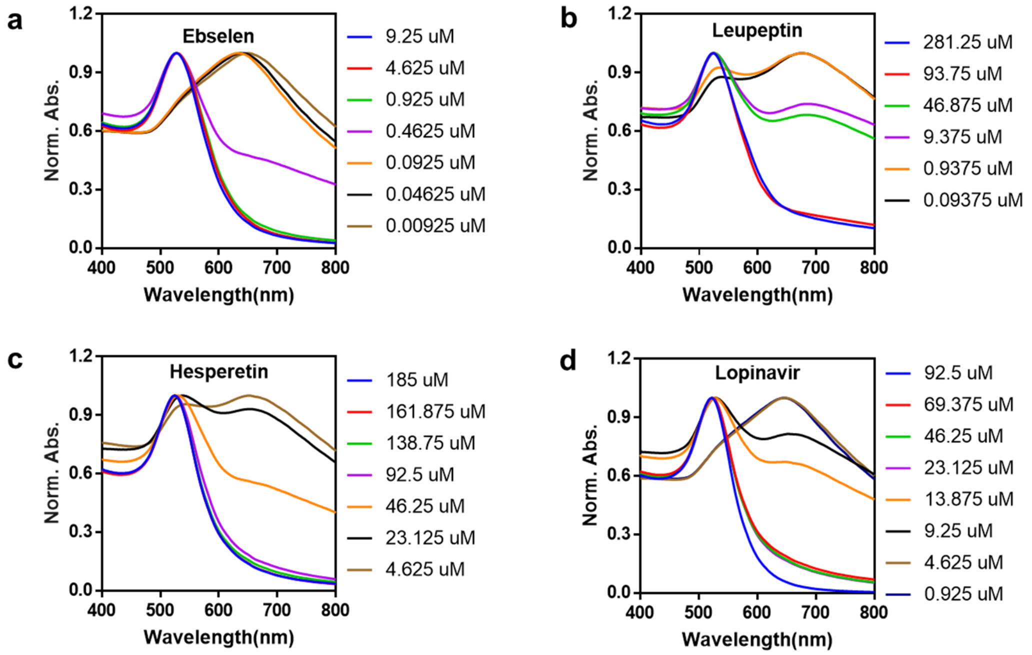

Supplementary Fig. 21. M^{pro} inhibition efficacy of drug candidates evaluated by using the MCAP-AuNP. UV-vis spectra of MCAP-AuNP solutions reacting with 18.5 nM of M^{pro} solutions preincubated with (a) ebselen, (b) leupeptin, (c) hesperetin, and (d) lopinavir. Before the M^{pro} reacted with MCAP-AuNP, M^{pro} was preincubated with each various concentration of inhibitor for 20 min, which inhibit its activity. After then, each inhibited state of M^{pro} was reacted with the MCAP-AuNP for 1h, and solutions were measured by UV-vis spectrometer. Note that the red shift of UV-vis spectra was increased as the concentration of the inhibitor was decreased, which indicated that the inhibition efficacy of each drug was represented by the peak shift of UV-vis spectra.

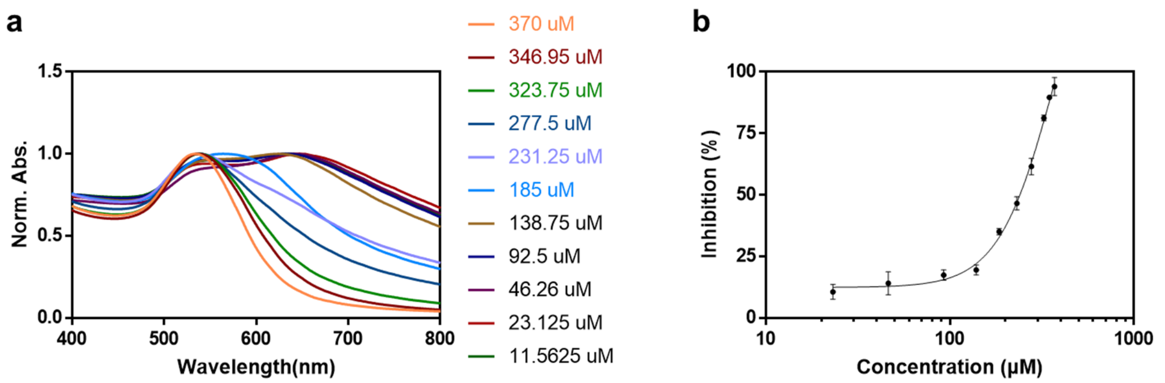

Supplementary Fig. 22. M^{pro} inhibition efficacy of hesperidin evaluated by using the MCAP-AuNP. (a) UV-vis spectra of MCAP-AuNP solutions reacting with each inhibited M^{pro} solution. Before the M^{pro} reacted with MCAP-AuNP, M^{pro} was preincubated with each concentration of hesperidin for 20 min, which inhibited its activity. After then, each inhibited state of each M^{pro} was reacted with the MCAP-AuNP for 1h. (b) The inhibition ratio of hesperidin for 1.4 nM of the M^{pro} depending on its concentration. Data are presented as mean values ± SD from n = 3 independent experiments. Error bars are not shown where they are smaller than the circle symbols.

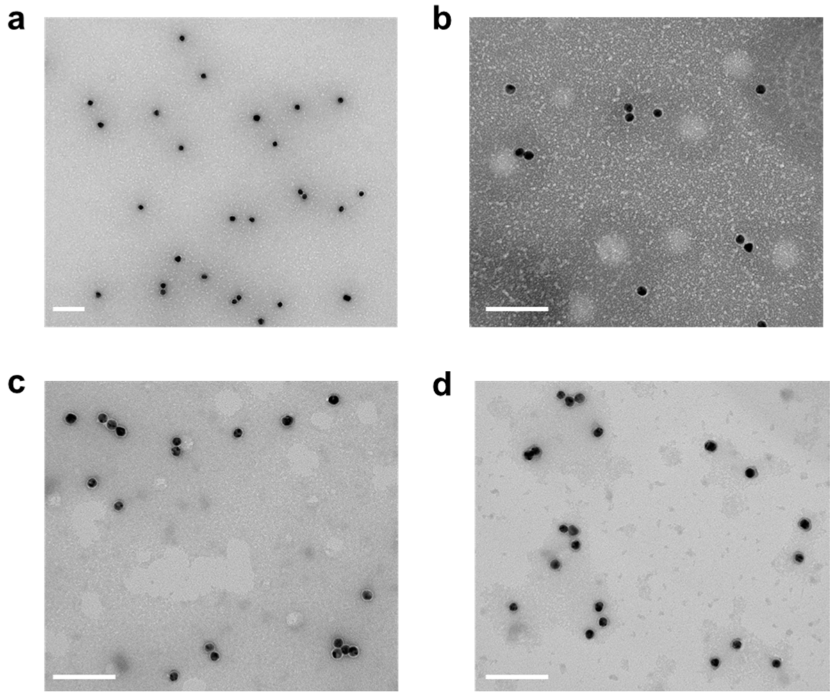

Supplementary Fig. 23. TEM image of the MCAP-AuNP solution reacted with inactivated M^{pro} solution. (a) TEM image of the MCAP-AuNP. The concentration of 0.1 mg mL^{-1} of M^{pro} solution was incubated with 1mM of (b) lopinavir, (c) leupeptin, and (d) hesperetin solution for 1h respectively. MCAP-AuNP solutions were reacted with each inactivated M^{pro} solution for 2h at 37 °C. The size of the white scale bar is 100 nm. The TEM images were measured at least three times.

Supplementary Table 1. Engineered amyloid sequences based on various protein-based amyloid-forming sequences and M^{pro}-cleavage sequence (L-Q↓(S, A, G)).

Name	Originated protein	Originated sequence	Engineered sequence
MCAP	Prion protein	GNNQQNY	LQGN**LQS**NQQNY
MCAP2	Prion protein	GNNQQNY	LQGN**LQA**NQQNY
MCAP3	Prion protein	GNNQQNY	LQGN**LQG**NQQNY
IAPP MCAP	IAPP protein	SNNFGAIL	SN**LQS**NFGAIL
Amyloid beta MCAP1	Amyloid beta protein	KLVFFAE	KL**LQS**VFFAE
Amyloid beta MCAP2	Amyloid beta protein	GGVVIA	GG**LQS**VVIA
Reposition MCAP	Prion protein	GNNQQNY	LQGNNQQNY**LQG**

Supplementary Table 2. Calculated results in comparison with the experimental values of potential inhibitors against the SARS-CoV-2 M^{pro}

Inhibitor	ΔG_{Dock} (kcal·mol^{-1})	K_i^{MD} (μM)	K_i^{Exp} (μM)
Ebselen	-6.6	14.51	0.23
Leupeptin	-6.0	39.95	16.69
Hesperetin	-7.3	4.45	25.19
Lopinavir	-6.4	20.34	6.25

Supplementary Table 3. Sigmoidal dose-response model of each inhibitor.

Inhibitor	Equation	R^2
Ebselen	Inhibition (%) = 0.95 + $\frac{97.80}{1+10^{Log0.39-[Ebselen]}}$	0.99
Leupeptin	Inhibition (%) = 2.29 + $\frac{103.61}{1+10^{Log28.57-[Leupeptin]}}$	0.91
Hesperetin	Inhibition (%) = 12.74 + $\frac{88.16}{1+10^{Log43.14-[Hesperetin]}}$	0.99
Lopinavir	Inhibition (%) = -3.432 + $\frac{100.40}{1+10^{Log10.7-[Lopinavir]}}$	0.96
