# Supplementary material for: Bioengineered amyloid peptide for rapid screening of inhibitors against main protease of SARS-CoV-2
Source: Nat Commun. 2024 Mar 7;15:2108. doi: 10.1038/s41467-024-46296-7 (PMC10920794; doi:10.1038/s41467-024-46296-7)
Supplement: Supplementary file 2 — Reporting Summary [file 41467_2024_46296_MOESM2_ESM.pdf]

nature portfolio

Corresponding author(s): Dae Sung Yoon

Last updated by author(s): Feb 10, 2024

Reporting Summary

Nature Portfolio wishes to improve the reproducibility of the work that we publish. This form provides structure for consistency and transparency in reporting. For further information on Nature Portfolio policies, see our Editorial Policies and the Editorial Policy Checklist.

Statistics

For all statistical analyses, confirm that the following items are present in the figure legend, table legend, main text, or Methods section.

n/a	Confirmed	
☐	☒	The exact sample size (*n*) for each experimental group/condition, given as a discrete number and unit of measurement
☐	☒	A statement on whether measurements were taken from distinct samples or whether the same sample was measured repeatedly
☒	☐	The statistical test(s) used AND whether they are one- or two-sided<br>*Only common tests should be described solely by name; describe more complex techniques in the Methods section.*
☒	☐	A description of all covariates tested
☒	☐	A description of any assumptions or corrections, such as tests of normality and adjustment for multiple comparisons
☐	☒	A full description of the statistical parameters including central tendency (e.g. means) or other basic estimates (e.g. regression coefficient) AND variation (e.g. standard deviation) or associated estimates of uncertainty (e.g. confidence intervals)
☐	☒	For null hypothesis testing, the test statistic (e.g. *F*, *t*, *r*) with confidence intervals, effect sizes, degrees of freedom and *P* value noted<br>*Give P values as exact values whenever suitable.*
☒	☐	For Bayesian analysis, information on the choice of priors and Markov chain Monte Carlo settings
☒	☐	For hierarchical and complex designs, identification of the appropriate level for tests and full reporting of outcomes
☒	☐	Estimates of effect sizes (e.g. Cohen's *d*, Pearson's *r*), indicating how they were calculated

Our web collection on statistics for biologists contains articles on many of the points above.

Software and code

Policy information about availability of computer code

Data collection	The UV-vis absorbance of the MCAP solution was measured by UV Express Version 4.1.3 provided by Perkin elmer in Lamda 365 and SoftMax Pro 7.1 provided by SoftMax Pro GxP Software<br>HRTEM images were measured by Windows® based SmartSEM™ control software equipped on a transmission electron microscope (SUPRA-55VP, Japan)<br>Zeta potential was measured by Zetasizer software 7.03 equipped on NanoZS Malvern instrument<br>XPS data was measured by Thermo avantage software® (V5.980) provided by Thermo scientific in K-alpha.<br>AFM images were measured by NX-10 (Park Systems, South Korea)<br>Photographs of the color changes of MCAP solutions were taken with Galaxy Note 20<br>Fluorescence intensity was measured by HIDEX software equipped on microplate reader (HIDEX plus, Japan)<br>Persistence lengths of each amyloid fibril were measured by Image J software in a TEM image.
Data analysis	Data were analyzed using Image J, Smart Scan®, Graphpad Prism v 7.0, Biovia Discovery Studio 2021 Client, UCSF Chimera version 1.14, and PyRx version 0.8.

For manuscripts utilizing custom algorithms or software that are central to the research but not yet described in published literature, software must be made available to editors and reviewers. We strongly encourage code deposition in a community repository (e.g. GitHub). See the Nature Portfolio guidelines for submitting code & software for further information.

Data

Policy information about availability of data

All manuscripts must include a data availability statement. This statement should provide the following information, where applicable:

- Accession codes, unique identifiers, or web links for publicly available datasets
- A description of any restrictions on data availability
- For clinical datasets or third party data, please ensure that the statement adheres to our policy

Source data are provided with this paper.
PDB accession codes: 6LU7 [https://doi.org/10.2210/pdb6LU7/pdb], 1R5B [https://www.rcsb.org/structure/1R5B], 6M5I [https://www.rcsb.org/structure/6M5I]
PubChem accession codes: 3194 [https://pubchem.ncbi.nlm.nih.gov/compound/3194], 72281 [https://pubchem.ncbi.nlm.nih.gov/compound/72281], 72429 [https://pubchem.ncbi.nlm.nih.gov/compound/72429], and 92727 [https://pubchem.ncbi.nlm.nih.gov/compound/92727].

Human research participants

Policy information about studies involving human research participants and Sex and Gender in Research.

Reporting on sex and gender	This information has not been collected
Population characteristics	This information has not been collected
Recruitment	This information has not been collected
Ethics oversight	This information has not been collected

Note that full information on the approval of the study protocol must also be provided in the manuscript.

Field-specific reporting

Please select the one below that is the best fit for your research. If you are not sure, read the appropriate sections before making your selection.

☒ Life sciences ☐ Behavioural & social sciences ☐ Ecological, evolutionary & environmental sciences

For a reference copy of the document with all sections, see nature.com/documents/nr-reporting-summary-flat.pdf

Life sciences study design

All studies must disclose on these points even when the disclosure is negative.

Sample size	At least 3 sample was conducted and sample size calculation is not required because it is a research design that has been experimented by collecting samples prospectively.
Data exclusions	No data was excluded from analysis.
Replication	All the attempts was repeated independently for at least three times for each sample tested. All replication of experiments was successful.
Randomization	All samples were randomly allocated into each experimental group
Blinding	All the investigators were blineded to groups allocation during data collection and analysis.

Reporting for specific materials, systems and methods

We require information from authors about some types of materials, experimental systems and methods used in many studies. Here, indicate whether each material, system or method listed is relevant to your study. If you are not sure if a list item applies to your research, read the appropriate section before selecting a response.

Materials & experimental systems

n/a	Involved in the study
☒	☐ Antibodies
☒	☐ Eukaryotic cell lines
☒	☐ Palaeontology and archaeology
☒	☐ Animals and other organisms
☒	☐ Clinical data
☒	☐ Dual use research of concern

Methods

n/a	Involved in the study
☒	☐ ChIP-seq
☒	☐ Flow cytometry
☒	☐ MRI-based neuroimaging
