# Supplementary material for: Bioengineered amyloid peptide for rapid screening of inhibitors against main protease of SARS-CoV-2
Source: Nat Commun. 2024 Mar 7;15:2108. doi: 10.1038/s41467-024-46296-7 (PMC10920794; doi:10.1038/s41467-024-46296-7)
Supplement: Supplementary file 4 — Description of Additional Supplementary Files [file 41467_2024_46296_MOESM4_ESM.pdf]

Supplementary Data 1

Description:
Input and output files of the AutoDock Vina calculations.
